# Drug-induced hypersensitivity syndrome related to piperacillin-tazobactam: a case report and review of the literature

**DOI:** 10.3389/fmed.2024.1338247

**Published:** 2024-03-28

**Authors:** Ya Shen, Shun-shun Cui, Xiao-bao Teng, Ming-feng Han

**Affiliations:** ^1^Department of Respiratory and Critical Care Medicine, Fuyang Infectious Disease Clinical College of Anhui Medical University, Fuyang, Anhui, China; ^2^Department of Respiratory and Critical Care Medicine, Fuyang People's Hospital, Fuyang, Anhui, China

**Keywords:** piperacillin-tazobactam, drug-induced hypersensitivity syndrome, drug reaction with eosinophilia and systemic symptoms, clinical features, prognosis

## Abstract

Allergic reactions to drugs caused by piperacillin-tazobactam are common in clinical practice. However, we also found a few cases of drug-induced hypersensitivity syndrome (DiHS)/Drug reaction with eosinophilia and systemic symptoms (DRESS) caused by piperacillin-tazobactam in our clinical work. We report a case of a 60-year-old female patient who was treated with piperacillin-tazobactam anti-infective therapy after the diagnosis of hematogenous lung abscess, developed fever, rash, and blood abnormalities after 26 days of application, and was later diagnosed as DIHS, which was improved after the administration of glucocorticoid and anti-allergic drugs. In addition, we also retrospectively analyzed 17 cases of DiHS caused by piperacillin-tazobactam from the PubMed databases between March 1980 and September 2023. The majority of the patients had an incubation period of more than 14 days, and the common clinical features included elevated eosinophil count/percentage, fever, rash, liver damage, and lymph node enlargement. After treatment with topical or systemic glucocorticoids, 16 of the 17 patients improved and one died because of the underlying condition. The clinical features of DiHS were diverse and included a long incubation period, skin rash, elevated eosinophils, and impaired organ function. Since some patients have atypical clinical features, clinicians should raise awareness of the disease, recognize these features early, and treat them promptly.

## 1 Introduction

Piperacillin-tazobactam is an antimicrobial agent commonly used in clinical practice that has strong antibacterial effects on anaerobic bacteria and a variety of gram-positive and gram-negative bacteria. Common adverse reactions include diarrhea, thrombocytopenia, anemia, itching rash, and fever (1). In recent years, several cases have been reported in which piperacillin-tazobactam caused drug-induced hypersensitivity syndrome (DiHS) (2–6).

Drug-induced hypersensitivity syndrome is a delayed-type IVb hypersensitivity reaction mediated by T cells (7), also known as drug reaction with eosinophilia and systemic symptoms (DRESS) syndrome, and the first case of DiHS caused by phenytoin was reported by Chaike in 1950 (8). The features of DIHS include fever, rash, elevated eosinophils, liver function impairment, and lymph node enlargement (9). DiHS can have a mortality rate of 10% and accounts for 10–20% of allergic reactions to drugs in hospitalized patients (10). The pathogenesis of DiHS is complex, and current research suggests that it is primarily associated with the reactivation of viruses, including HHV-6, EBV, HHV7, and cytomegalovirus (11, 12). The Japanese diagnostic criteria for DiHS also included reactivation of HHV-6 as diagnostic criteria (13).

Therefore, to improve clinicians’ awareness of DiHS caused by piperacillin-tazobactam, we performed a retrospective analysis of one patient confirmed in our hospital and 17 patients from a literature review, this is the largest sample size study on this disease related to piperacillin-tazobactam. We hope to help clinicians further understand the clinical characteristics of such patients, identify them early, standardize their treatment promptly, and thus improve their prognosis.

## 2 Case presentation

**Figure 1:**
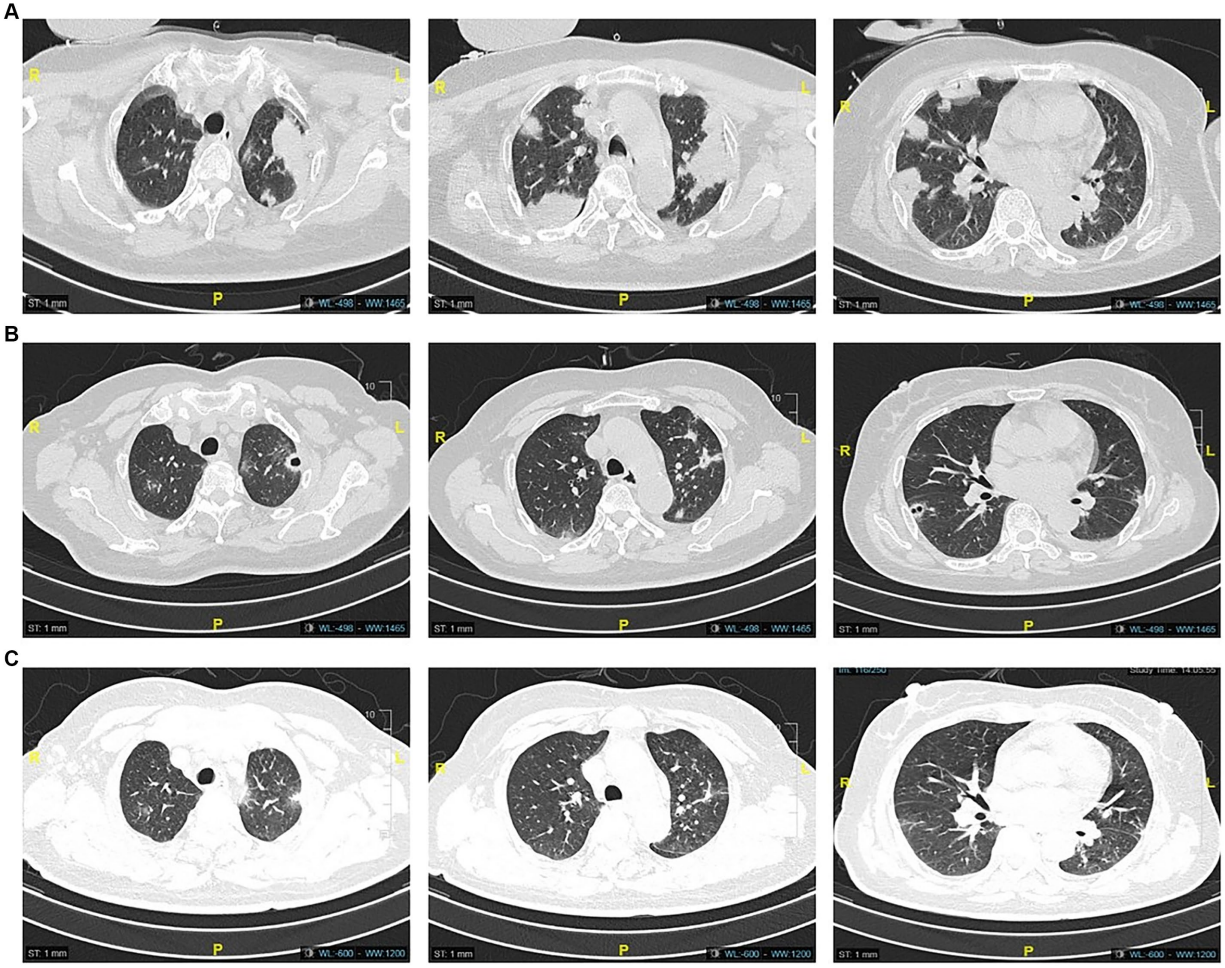
Computed tomography images on admission showed multiple mass shadows **(A)**, and CT images after 15 days of piperacillin-tazobactam anti-infection treatment showed partial resorption of the lung lesions in comparison **(B)**. The lesions continued to resorb after 30 days **(C)**.

A 60-year-old female was admitted to the hospital on August 1, 2022, due to 1 week of recurrent fever with progressive dyspnea, with a previous history of type 2 diabetes. Her chest CT showed multiple masses and cavity changes in both lungs (Figure 1A), and the blood culture showed bilateral aerobic bottle growth of *Klebsiella pneumonia*. A diagnosis of a hematogenous pulmonary abscess was made. The drug sensitivity results of blood culture suggested sensitivity to piperacillin-tazobactam, which was then administered as an anti-infective agent.

**Figure 2:**
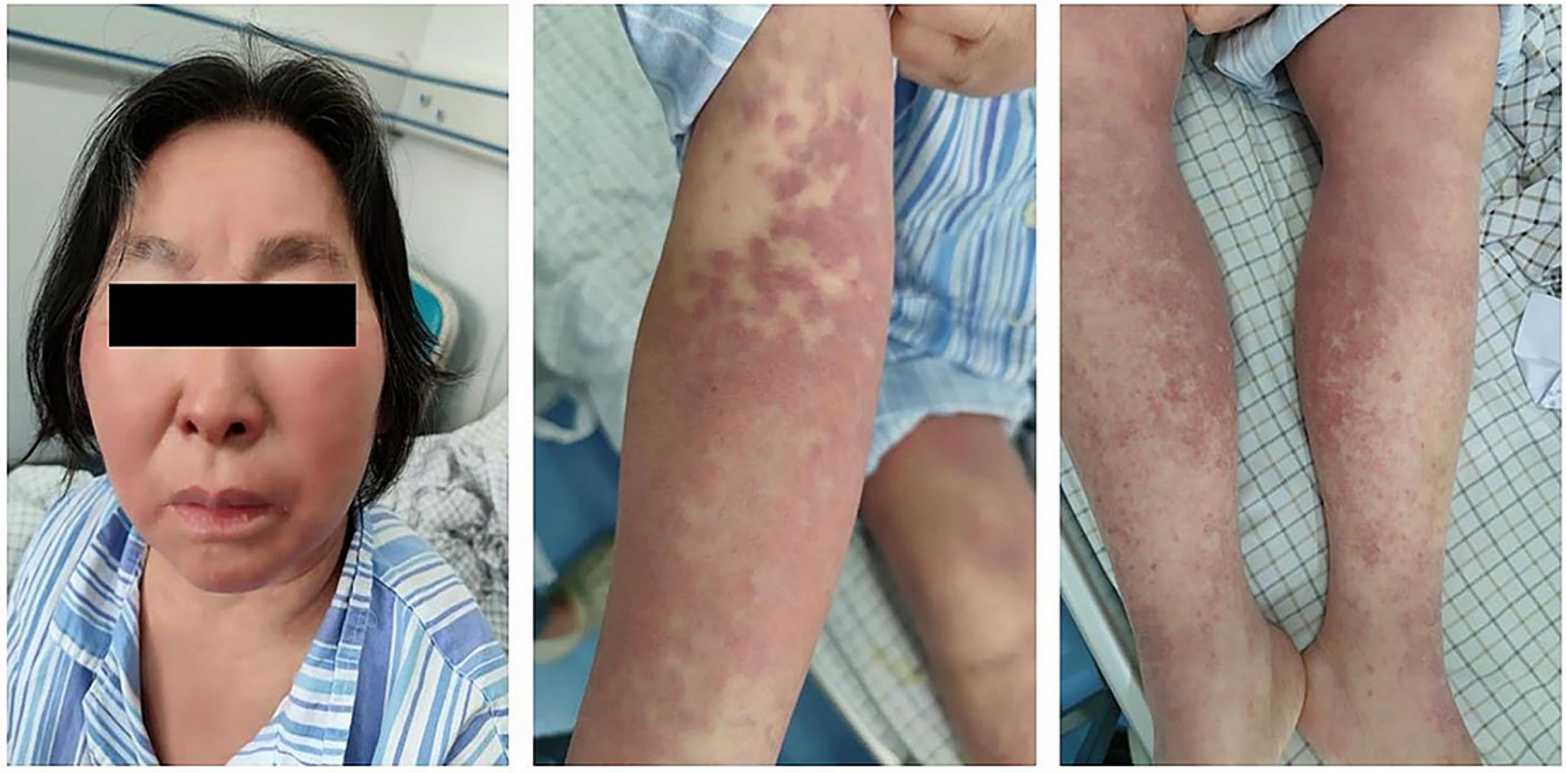
Facial edema and generalized (face, limbs, and chest) skin rash in our patient.

**Figure 3:**
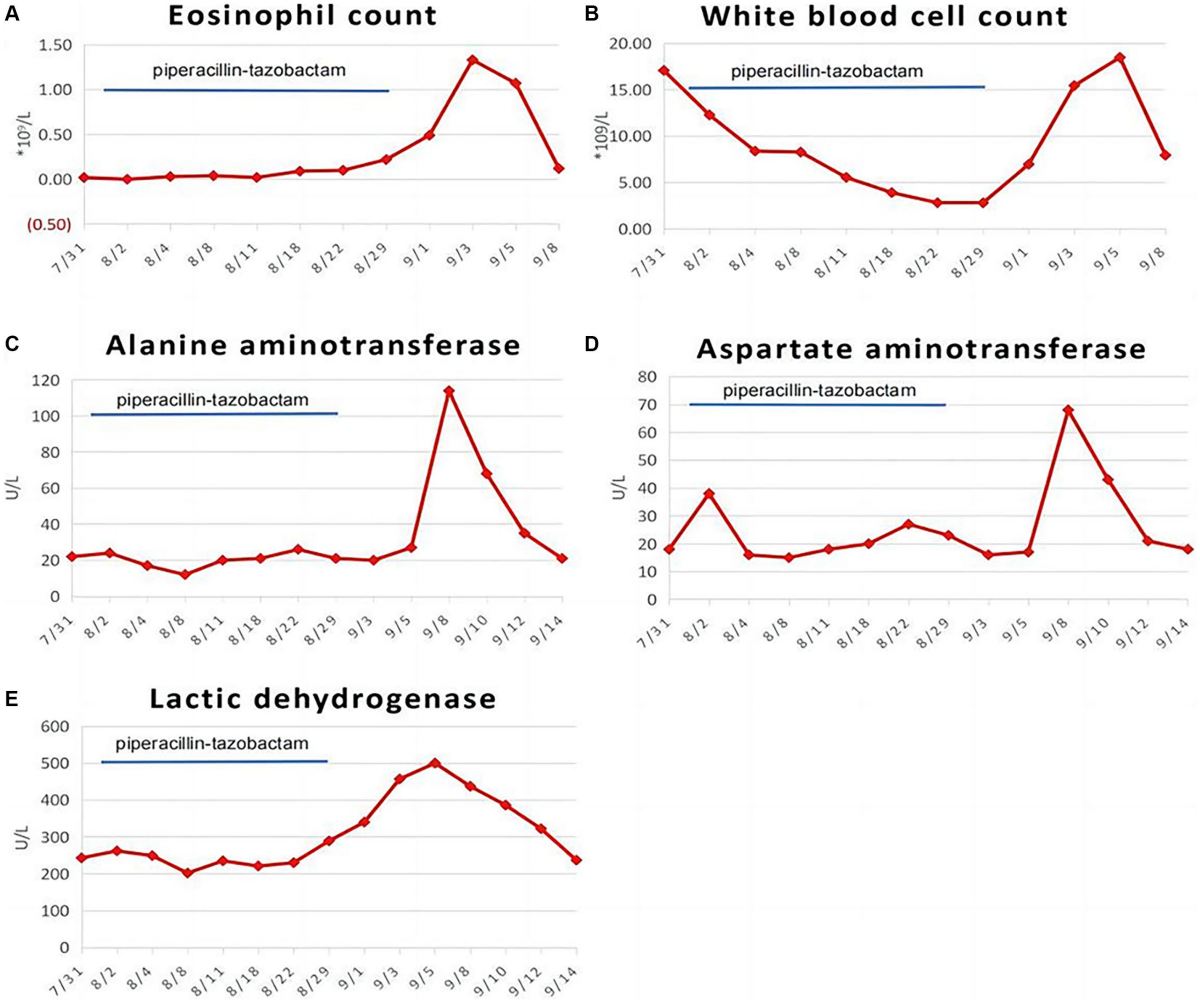
Dynamic changes of eosinophil count **(A)**, white blood cell count **(B)**, alanine aminotransferase **(C)**, aspartate aminotransferase **(D)**, and lactate dehydrogenase **(E)** during hospitalization in our patient.

During the treatment period, the patient’s symptoms gradually improved, and the lesion was partially absorbed on Day 15 upon review of the chest CT (Figure 1B). On Day 26, the patient developed a low fever, with her temperature reaching 37.8°C. Three days later, she began to develop a punctate red rash in the front chest and forearm of both upper limbs with pruritic discomfort (Figure 2). We could not rule out delayed drug allergy after a dermatology consultation, so we discontinued the use of piperacillin-tazobactam. The range of the posterior rash expanded to the face and lower limbs (Figure 2), the occipital and mandibular lymph nodes became enlarged, and the body temperature increased to a maximum of 39.5°C. A repeat chest CT showed continued improvement of the lesion (Figure 1C). Fever-related tests, such as pneumonia pathogen detection, rheumatoid immune complexes, and ferritin, were completed, and all results were negative. The patient had a gradual rise in eosinophils to 1.33*109/L, leukocytes to 18.47*109/L, and lymphocytes to 7.70*109/L, as well as a significant increase in abnormal lymphocytes (7%) accompanied by abnormal liver function, with an ALT level of 114 U/L, AST level of 68 U/L, and a gradual rise in LDH to 500 U/L (Figure 3).

Combining the clinical manifestations and the laboratory findings, the patient was considered to have DiHS caused by piperacillin-tazobactam, with a RegiSCAR score of 8 (14). The patient was treated with systemic glucocorticoids and oral anti-allergy drugs. On September 8, the fever stopped, the rash gradually subsided, and the relevant laboratory indices decreased upon review. Three months later, we followed up on the patient’s discharge by phone, and she said that she had not experienced any further discomfort after discharge and that her chest CT and laboratory test indexes had returned to normal.

## 3 Discussion

CABANAS reported the first case of DiHS associated with piperacillin-tazobactam in 1998 (15). The pathogenesis of this disease has not yet been fully elucidated. Recent reviews have pointed out that DiHS/DRESS caused by some anti-epileptics, anti-gout, antibiotics, and anti-viral drugs are associated with genetic polymorphisms in the HLA allele, and that mechanisms such as specific signaling pathways in T-cell activation, production of cytokines and chemokines, and viral reactivation also play a role in the pathogenesis of DiHS/DRESS (16, 17). Rutkowski reported six patients with piperacillin-tazobactam-induced DiHS, and three patients were found to be HLA-B62-positive by testing for HLA typing (6). Therefore, we also performed HLA-B62 on our patient, and the results were negative.

In terms of clinical symptoms, the patient first presented with fever followed by generalized rash and lymph node enlargement. The rash manifestations are always diverse, including maculopapular and bryoid rash, exfoliative dermatitis, urticaria, purpura, eczema, and impetigo (9). The patient we reported mainly showed red maculopapular rashes. Unlike an allergic drug rash, the rash caused by DiHS often does not subside quickly after discontinuation of the drug and may even continue to worsen.

In addition to elevated eosinophils and liver function impairment, there was also elevated lactate dehydrogenase (LDH) in the laboratory findings in our patient. We conducted myocardial zymography, brain natriuretic peptide (BNP), cardiac color ultrasound, and other examinations, and all the results showed no abnormalities. Some studies have shown that the most common organ function damage caused by DiHS is liver damage, followed by renal, pulmonary, and cardiac damage. Cardiac damage can manifest as chest pain, tachycardia, dyspnea, hypotension, etc., and the appearance of these clinical signs may occur long after the rash has subsided (18), so this patient needs to be further followed up for the development of cardiac and other organ damage. Considering that the lymphocyte count was significantly higher in the patient, we further completed the blood cell classification test, which showed elevated abnormal lymphocytes and monocytes. This led us to consider whether the patient was complicated with EB virus infection, but the patient’s EBV-DNA result was negative, which was rather unexpected.

To further understand the clinical features and diagnosis and treatment of DiHS due to piperacillin-tazobactam. We searched the relevant articles of the PubMed database from March 1980 to September 2023, using “Piperacillin-tazobactam, Drug-Induced Hypersensitivity Syndrome (DiHS), Drug Reaction With Eosinophilia And Systemic Symptoms (DRESS)” as the search terms, and five articles were included. A total of 17 patients were eligible for DiHS caused by piperacillin-tazobactam. The general information, clinical symptoms, laboratory results, and outcomes of the 17 patients and our patient were summarized (Table 1). After analyzing the case data, we found the same proportion of men and women. The mean age was 52.94 years, ranging from 4 to 83 years, with nine patients over the age of 60 years, suggesting that the population affected by this disease might be mainly middle-aged and elderly. The mean incubation period of 18 patients was 18.9 days, with 15 patients having an incubation period longer than 14 days. All patients developed rashes of varying degrees at the early stage of the disease, indicating a relatively high incidence of rash in this disease. The 11th patient had AGEP prior to DRESS. Despite improvement in AGEP symptoms, the patient died as a result of the prior condition and the development of DRESS (5).

**Table 1:** Clinical features, laboratory outcomes, treatment strategies, and outcomes of the 18 patients.

Patients	Age (years)/gender	Incubation (d)	Skin Rash	Enlarged lymph nodes	Highest temperature (°C)	Eosinophil count/Percent (*10^9^/L/%)	ALT (U/L)	AST (U/L)	Diagnostic score	Outcome
_1_(2)	4/F	14	Yes	Yes	40.0	2.94	>500	>400	6^a^	Improved
_2_(3)	43/F	21	Yes	Yes	38.5	1.05	108	66	7^a^	Improved
_3_(4)	61/M	14	Yes	No	38.8	5.90	21	28	5^a^	Improved
_4_(4)	67/F	24	Yes	Yes	38.5	1.13	102	69	7^a^	Improved
_5_(4)	83/M	28	Yes	No	37.2	3.25	104	90	6^a^	Improved
_6_(4)	77/M	14	Yes	No	38.5	1.30	105	36	5^a^	Improved
_7_(4)	59/M	21	Yes	No	38.6	1.10	117	59	5^a^	Improved
_8_(4)	60/M	20	Yes	Yes	38.9	0.80	240	161	7^a^	Improved
_9_(4)	39/F	8	Yes	Yes	40.0	2.60	13	22	6^a^	Improved
_10_(4)	48/M	28	Yes	No	38.0	13.1%	1,242	881	5^a^	Improved
_11_(5)	74/F	9	Yes	No	38.5	36%	64	90	5^b^	Dead
_12_(6)	61/F	31	Yes	-	39.2	2.68	27	-	7^a^	Improved
_13_(6)	29/M	18	Yes	-	39.7	1.64	30	-	4^a^	Improved
_14_(6)	54/F	4	Yes	-	39.8	0.90	39	-	4^a^	Improved
_15_(6)	12/M	14	Yes	-	40.0	0.79	1,099	-	6^a^	Improved
_16_(6)	69/F	18	Yes	-	38.3	1.47	52	-	5^a^	Improved
_17_(6)	53/M	25	Yes	-	39.8	2.60	212	-	8^a^	Improved
18^case^	60/F	29	Yes	Yes	39.5	1.33	114	68	8^a^	Improved

^a^RegiSCAR scoring system (Total score < 2 excluded, 2–3: possible, 4–5: probable, and >5: definite) (14).

^b^Japanese Consensus Group Criteria (five criteria: atypical; seven criteria: typical) (13).

Only one patient had no febrile manifestations, while the rest had moderate or higher fever (>38°C), and all patients had increased eosinophil counts/percentage, further confirming that DiHS is an allergy-mediated disease. Li et al. (19) retrospective analysis of 104 DiHS patients revealed that 71.2% of patients presented with lymph node enlargement at more than two sites, 86.5% had fever, and 69.2% had eosinophilia. This result suggests a lower proportion of patients with eosinophilia and a higher proportion of patients with enlarged lymph nodes. Of course, their study subjects included all patients with drug-induced DiHS, whereas our subjects were limited to patients with DiHS due to piperacillin-tazobactam, and our small sample size may be biased. Only five out of 18 patients had normal liver function and the rest had elevated liver enzymes (ALT/AST), with more than half of the patients having liver enzymes more than twice the upper limit of normal values. Kuchinskaya et al. (20) retrospectively analyzed the clinical characteristics of 16 patients with DiHS, and the results showed that all the patients had liver impairment, and no other organ impairment was seen in any patient, which is generally consistent with our findings.

All patients discontinued piperacillin-tazobactam immediately after the diagnosis of DIHS, nine received topical glucocorticoids, 13 received systemic glucocorticoids, and a small number of patients received antihistamines. Seventeen of the 18 patients improved after treatment. Therefore, in terms of treatment strategy, the priority is to discontinue piperacillin-tazobactam while symptomatic treatment is provided for symptoms such as fever and rash. In recent years, many studies have shown that glucocorticoids play an important role in DIHS. Mizukawa et al. established a DiHS severity scale based on clinical data from 55 patients with DiHS and guided the application of glucocorticoids based on patient scores (21). Another study applied immunosuppressants to patients with DiHS, but the exact effect remained unclear due to the small sample size (22).

## 4 Conclusion

Patients with DiHS due to piperacillin-tazobactam generally have a long incubation period and clinical features that include a generalized rash, fever, elevated eosinophils, and hepatic impairment, and generally have a good prognosis if treated promptly. This requires that clinicians pay attention to adverse drug reactions while applying piperacillin-tazobactam, and if signs and symptoms similar to those associated with DiHS appear, a clear diagnosis should be made and reasonable treatment should be administered promptly. At the same time, the treatment of underlying and co-morbid diseases is also crucial.

## Data availability statement

The original contributions presented in the study are included in the article/supplementary material; further inquiries can be directed to the corresponding author.

## Ethics statement

Written informed consent was obtained from the individual(s) for the publication of any potentially identifiable images or data included in this article. Written informed consent was obtained from the participant/patient(s) for the publication of this case report.

## Author contributions

YS: Writing – original draft. S-sC: Writing – original draft. X-bT: Writing – original draft. M-fH: Writing – review & editing.
